# Clinical and Radiological Outcomes After Isolated Anterior Horn Repair of Medial and Lateral Meniscus at 24 Months’ Follow-up, With the Outside-In Technique

**DOI:** 10.7759/cureus.17917

**Published:** 2021-09-12

**Authors:** Vasilios Raoulis, Apostolos Fyllos, Christos Baltas, Philipp Schuster, George Bakagiannis, Aristeidis H Zibis, Michael Hantes

**Affiliations:** 1 Anatomy Lab, Department of Medicine, School of Health Sciences, University of Thessaly, Larissa, GRC; 2 Department of Orthopedic Surgery & Musculoskeletal Trauma, University General Hospital of Larissa, Larissa, GRC; 3 Centre of Sports Orthopedics and Special Joint Surgery, Orthopedic Hospital Markgroeningen, Markgroeningen, DEU; 4 Department of Orthopedics and Traumatology, Paracelsus Medical Private University, Clinic Nuremberg, Nuremberg, DEU

**Keywords:** meniscus surgery, meniscus anterior horn radiological anatomy, meniscus repair, meniscus outside-in, anterior horn meniscus

## Abstract

Background

The effects of repair of isolated anterior horn meniscus lesions have not been thoroughly described in the literature. We aimed to evaluate outcomes with subjective clinical scores and imaging modalities after repair of isolated anterior horn tears, at 24 months’ follow-up.

Methods

Records of all patients that opted for surgical repair of isolated, anterior horn tears of the medial and lateral meniscus were retrospectively reviewed, between 2016 and 2018. All patients were treated with arthroscopic outside-in technique by the same surgeon. Preoperative and postoperative clinical files were accessed to recover records of preoperative symptomatology, patient-reported scores [International Knee Documentation Committee (IKDC) rating, Lysholm score and Tegner activity level], preoperative and postoperative MRI data and time from injury to surgery.

Results

Mean age of eight patients was 25.25 years (range 18-37 years). Diagnostic preoperative MRI revealed isolated anterior horn tear of the lateral meniscus and medial meniscus in five patients and an isolated anterior horn tear of the medial meniscus in three patients. Mean time from injury to surgical repair was 23.75 days (range 7-43). We considered seven out of eight repairs to be successfully healed. At 24 months’ follow-up: Mean Lysholm score was 92.25 (range 89-95), Tegner activity scale score was 6.5 (range 5-8) and IKDC score was 91.78 (range 87.8-94.4). All scores significantly improved compared to preoperative values (p<0.001).

Conclusions

Outside-in is a reliable technique to repair meniscal anterior horn tears, both medially and laterally, with high healing rates and patient satisfaction in young, active patients.

## Introduction

The recommended and validated classification of meniscal injury consists of three circumferential (assessing vascularity) and three radial zones (determining anteroposterior location) [1-4]. Medial and lateral anterior horn meniscal injuries, outcomes and recommendations are seldom reported as a separate entity in literature. Approximately 15% of athletes with acute knee trauma and hemarthrosis are prone to isolated meniscus tears, and a higher ratio of medial (69%-76%) to lateral (24-31%) meniscus tears has been observed [5,6]. Isolated anterior horn tear is a rare entity, comprising of 1.6-3.5% of all meniscus injury [4,5,7,8] or 8% as a concomitant knee injury [9].

The medial meniscus is tightly adherent to the joint capsule. The anterior third is smaller, approximately one-third to one-half of the size of the posterior third. In a human cadaveric study, the applied load on the medial compartment was evenly distributed between meniscus and tibial plateau, whereas the majority of the load on the lateral side was carried by the meniscus [10]. The lateral meniscus is smaller than the medial meniscus and is not as firmly attached to the tibial plateau allowing greater mobility. This advantage allows lateral meniscus to be more forgiving to stress, explaining firstly low tear incidence and secondly it could act as a protection of meniscal repair [11,12]. The anterior and posterior thirds of the lateral meniscus are of equal size [13,14]. The lateral meniscus supports a larger percentage of load-bearing than the medial meniscus and more specifically the anterior horn [10,15-17]. Finally, the insertion of the anterior horn of the lateral meniscus overlaps with the tibial anterior cruciate ligament (ACL) insertion in both the coronal and sagittal planes, and can become impinged between the lateral femoral condyle and the anterior edge of the lateral tibial eminence, often producing a macerated, degenerative-like tear morphology [18].

The effects of surgical repair of isolated injury to the anterior horn of either meniscus have not been thoroughly described in the literature. The aim of the present study was to evaluate outcomes with subjective clinical scores and imaging modalities after repair of isolated anterior horn tears of the medial or lateral meniscus with the outside-in technique.

## Materials and methods

Following Institutional Scientific and Ethics Review Board approval (14/01/20, protocol number 865), we retrospectively reviewed records of all patients that opted for surgical repair of traumatic, isolated, anterior horn tears of the medial and lateral meniscus, between 2016 and 2018.

Patients with a diagnosis of an isolated tear of the anterior horn of the lateral or medial meniscus, based on clinical and MRI findings and confirmed intraoperatively, were included in the study. Exclusion criteria were concomitant meniscal, ligamentous or osseous injury, patients under the age of 17, and patients that opted for conservative management. Preoperative and postoperative outpatient files were accessed to recover records of preoperative symptomatology, patient-reported scores [International Knee Documentation Committee (IKDC) rating, Lysholm score and Tegner activity level], MRI data and time from injury to surgery. Surgical records were accessed to recover number of sutures placed and operation duration.

All arthroscopic repairs were performed by the same surgeon, with the same instrumentation and with the use of the “outside-in” arthroscopic technique. 

Surgical technique

**Figure 1:**
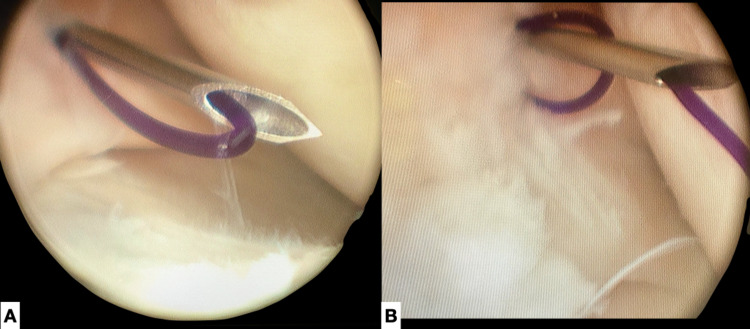
Outside-in technique. A. Arthroscopic image of the first needle, loaded with the PDS suture. The free ends of the suture are tied together. B. Arthroscopic image of suture passing through the previously created loop, with gentle manipulation of the two needles.

**Figure 2:**
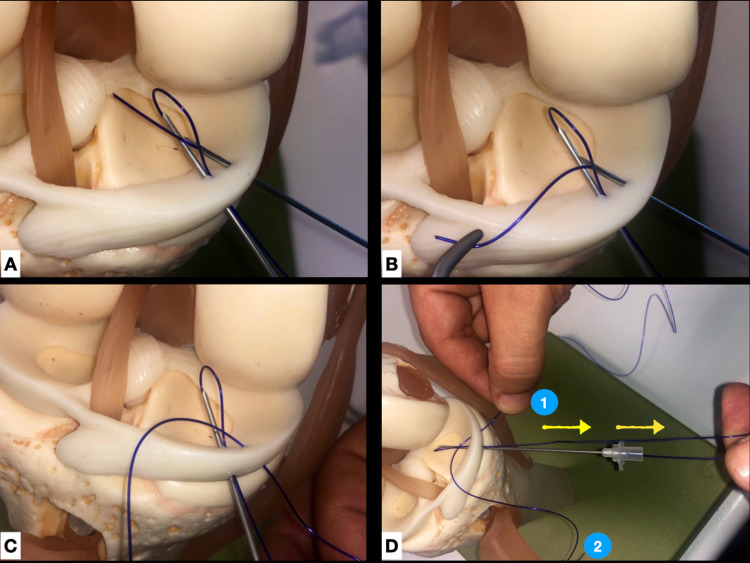
Outside-in technique. A. Demonstration of suture passing through the loop on anatomic high-fidelity training model. B, C. Grasping of suture free end and pulling outside the joint. D. While holding the first free end of the second suture (1), the other free end of the same suture (2), lies free outside the joint (previously pulled through the arthroscopic portal). The loop created by the first suture is pulled outside the joint (arrows), along with the other suture.

A typical knee arthroscopy was performed with two anterior portals (anterolateral and anteromedial). With the arthroscope placed in the ipsilateral portal, the patient’s knee is flexed 90^0^-100^0^ for medial anterior horn tears or placed in “figure-of-four” position for lateral anterior horn tears. The meniscus tear edges are rasped sequentially. A No1 PDS suture is passed through two 18-gauge needles. In the first needle, the two free-ends of the PDS suture are tied together with a knot. The same (first) needle, under arthroscopic guidance, is inserted until it crosses the tear site in a way that the suture does not interfere with the sharp end of the needle. The second needle with the loaded PDS suture is inserted until it emerges through the meniscus on the opposite side of the tear from the first needle. With the help of a grasper or with gentle manipulation, the suture from the first needle is pulled out so that a suture loop is visible from the arthroscope. The second needle with the loaded suture is passed through the loop and this suture is then retrieved and pulled out through the contralateral portal. Finally, by placing a finger on the suture whose free ends are tied together, the suture is pulled out, while holding the end of the second one firmly (Figures 1, 2). A small skin incision is made between the sutures and dissection is carried out down to the capsule. Τhe reduction of the meniscus tear is arthroscopically confirmed and a large extra-articular knot is tied directly against the capsule, with caution in medial anterior horn meniscus tears so as not to include the infrapatellar branch of the saphenous nerve in the knot. Sutures are placed until complete opposition of the two sides of the tear.

Rehabilitation protocol

Postoperatively, a standardized rehabilitation protocol was initiated for all patients. This comprised of no weight-bearing for the first two weeks, with knee brace locked in extension, and isometric quadriceps exercises were encouraged. This was followed by partial weight-bearing with the use of crutches for another five weeks. During this second stage, passive knee flexion was permitted from 0-90^0^. After four weeks, an intensive knee strengthening regime was prescribed for all patients, and they were instructed to avoid activities that would produce increased axial loading of the knee joint (like jumping or running) for at least four months. Sports participation was allowed after the six months’ follow-up consultation.

Follow-up protocol

Follow-up consultations were made every two weeks for the first two months, and then at three months, six months, 12 months postoperatively and yearly thereafter. Residual joint line pain, joint effusion, locking or pain during meniscal provocation tests were recorded at six, 12 and 24 months follow-ups, as presented by Barrett et al [19]. According to the criteria of Barrett et al, an unhealed meniscus was defined when any one of the symptoms or physical signs (swelling, joint-line tenderness, locking or blocking, and a positive McMurray test) were present. Absence of the four signs suggested a completely healed meniscus. Patient-reported outcomes were assessed with the International Knee Documentation Committee (IKDC) rating, Lysholm score and Tegner activity level at the 24 months’ follow-up [20]. For the 12months follow-up consultation, an MRI was requested.

MRI protocol

All MR imaging examinations were performed at 1.5T scanner (PHILIPS, NL) and for the purposes of the present study, the most frequent sequences used were sagittal short TI inversion recovery (Sag STIR), sagittal proton density (Sag PD), sagittal transverse relaxation (Sag T2*), coronal longitudinal relaxation (Cor T1) and axial STIR (Ax STIR). The meniscal lesions were graded on MRI on a scale of 0-3 (0: low signal intensity; 1: irregularly marginated intrameniscal signal; 2: linear signal not extending to articular surface; 3: linear signal intensity extending to surface) [21].

Statistics

Descriptive statistics and Student’s t-test for paired samples were used to compare preoperative and postoperative patient-reported outcomes. Statistical significance was set as p<0.05.

## Results

Eight patients (seven male patients and one female) fulfilled the inclusion criteria. Mean age of eight patients was 25.25 years (range 18-37 years, SD=5.38). Mechanism of injury was high energy sports activity (football) in six patients, fall from height (stairs) in one patient and traffic accident injury for one patient. Diagnostic preoperative MRI revealed isolated anterior horn tear of the lateral meniscus in five patients and an isolated anterior horn tear of the medial meniscus in three patients. Tear morphology was arthroscopically verified. There were six vertical tears, two in red-red zone and four in the red-white zone. The remaining two patients suffered macerated tears. Preoperatively, all patients had pain with full knee extension and pain during athletic activities. No effusion, clicking or positive meniscal provocation tests were documented. Preoperative mean Lysholm score was 75.25 (range 70-80, SD 2.68), Tegner activity scale score was 4.37 (range 3-5, SD 0.7) and IKDC score was 71.99 (range 66.7-75.9, SD 3.2). Mean time from injury to surgical repair was 23.75 days (range 7-43, SD 11.13). Perioperatively, three patients required two sutures and five required three sutures (all three medial tears required three sutures) to completely seal the meniscal tear gap. Mean operation duration was 43.2 minutes (range 35-54, SD 4.1)

Immediately postoperatively, one patient complaint of subcutaneous knot irritation by palpation (without radiating pain or paresthesia) that resolved by the six months’ outpatient follow-up without intervention. No other perioperative or postoperative complications were observed.

24 months’ follow-up: Mean Lysholm score was 92.25 (range 89-95, SD 2.17), Tegner activity scale score was 6.5 (range 5-8, SD 0.87) and IKDC score was 91.78 (range 87.8-94.4, SD 2.58).

**Figure 3:**
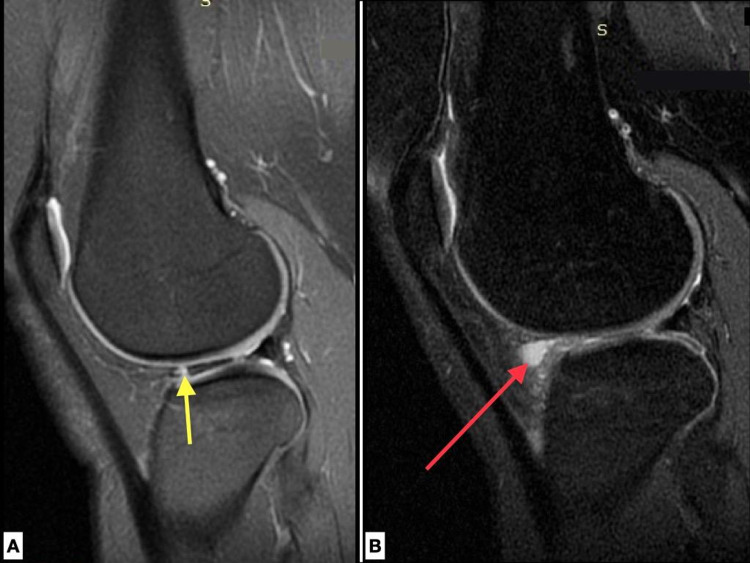
MRI pre-op and post-op. A. T2 sagittal image of the right knee of a 22-year-old female patient with a tear (yellow arrow) in the anterior horn of the lateral meniscus. B. STIR sagittal image of the same patient 12 months postoperatively, where a meniscal cyst is formed (red arrow).

**Figure 4:**
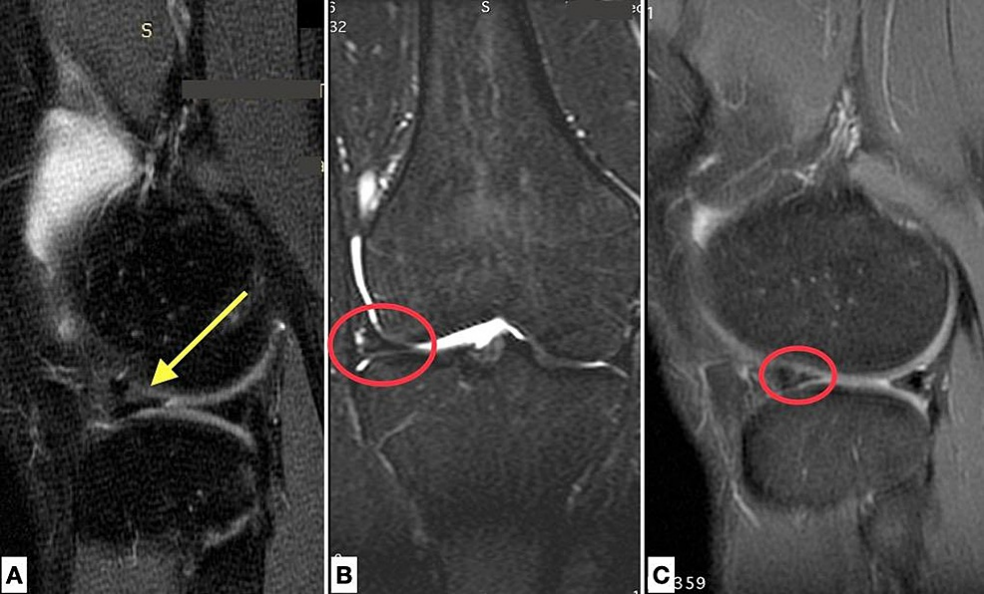
MRI pre-op and post-op. A. Sagittal PD FSE image of the right knee of an 18-year-old male patient with a tear (yellow arrow) in the anterior horn of the lateral meniscus. B. Coronal STIR and C. Sagittal PD FSE image of the same patient 12 months postoperatively, depicting a healed meniscus (red cycle) with signal grade 1.

**Figure 5:**
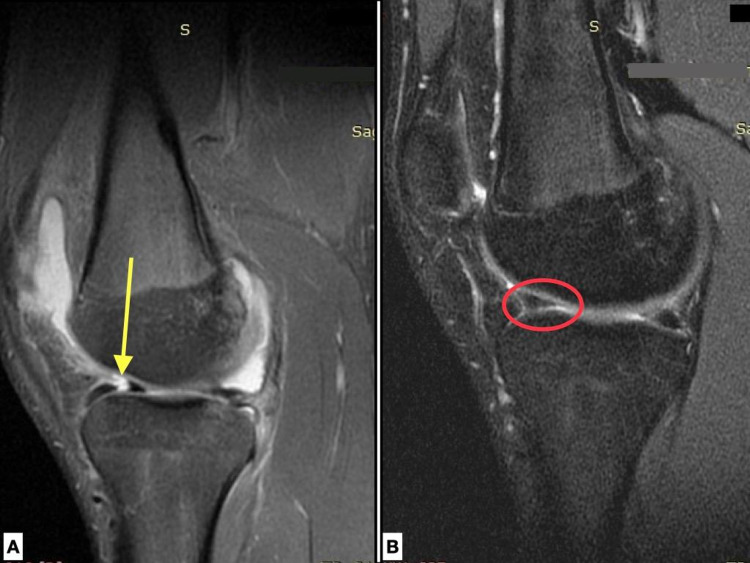
MRI pre-op and post-op. A. Sagittal PD FSE image of the left knee of an 18-year-old male patient with a tear (yellow arrow) in the anterior horn of the lateral meniscus. B. Sagittal PD FSE image of the same patient 12 months postoperatively with a healed meniscus (red cycle).

Patients reported significantly improved Lysholm, IKDC and Tegner scores at 24 months’ follow-up, compared to preoperative (p<0.001) (Table 1). None of the aforementioned clinical signs and symptoms as published by Barrett et al were present in any patient during 24 months of follow-up. On postoperative MRI, no meniscal extrusion or gap was present. Furthermore two patients had grade 1 meniscal lesion, three patients had grade 2, one patient had grade 3, and one patient could not be graded due to the presence of a large enclosed postoperative meniscal cyst (Figures 3, 4, 5).

**Table 1:** Patients scores. Comparison of mean patient-reported scores preoperatively and 24 months postoperatively. IKDC: International Knee Documentation Committee.

Score	Preoperative	24 months	sig.
Lysholm	75.25	92.25	p<0.001
Tegner	4.37	6.5	p<0.001
IKDC	71.99	91.78	p<0.001

## Discussion

This is a case series study of eight young patients with isolated medial or lateral anterior horn tears that were successfully and acutely repaired with the outside-in arthroscopic technique. According to clinical tests and imaging results at 24 months’ follow-up, seven out of eight repairs were evaluated as healed. The MRI of one asymptomatic, satisfied patient showed meniscal cyst development and consequently was considered unhealed. 

Anterior horn meniscal injury is a rare entity and only three case series with small samples have been previously published, focusing on lateral meniscus [8,18,22]. Choi et al used all-inside technique in seven young male footballers, with return to sport at six months after surgery. However, no patient-reported outcomes or imaging data were presented [22]. Hagino et al reported great improvement in mean Lysholm score 12 months postoperatively, similar to our results in 8 young male footballers with anterior horn tears of the lateral meniscus, although treatment modality was not consistent (only two tears were repaired, the rest were partially excised) [8]. In the study by Zhen et al. which focused on anterior horn tears of the lateral meniscus, patients with anterior horn tears were organized based on tear morphology [18]. The macerated tear type was present in the majority of patients (42%), the other three being horizontal tears, vertical tears or complex tears. The macerated tear group showed significantly less functional recovery of Lysholm and IKDC scores compared to other groups. Moreover, another group was studied with preoperative anterior horn cysts, that showed even worse patient-reported outcomes. At the last follow-up (range 15-36 months), Lysholm and IKDC scores were 84.4 (range, 78-90 points) and 80.3 points (range 74-90 points), respectively, for patients with macerated type of tear [18]. Recently, it was reported that patients who underwent acute meniscal repair (<6 weeks from injury) demonstrated significantly higher improvements on the Tegner activity scale and Lysholm score compared with patients treated beyond six weeks from injury, regardless of the meniscal tear zone, at a minimum follow-up of two years. Unfortunately, radial location of meniscal tears was not separately reported [23].

Among the commonly used techniques for meniscal repair (inside-out, outside-in, and all-inside techniques), the outside-in repair technique is ideal for anterior horn tears because it allows for adequate access to the anterior horn of the meniscus, provides a stable fixation construct, and has been appropriately modified to avoid leaving prominent intra-articular material [24]. With this technique, inexpensive spinal needles and sutures can be used without the need for additional implants or instruments. This is ideal in smaller knees and knees with tight compartments. Saphenous nerve injury (main branch or infrapatellar branch) and infection are seldom seen complications [25].

Early evaluation of the meniscal healing status after repair is important for the surgeon to inform the patients whether they can increase their activities or return to sports [19,26]. Clinical assessment can lead to false-positive or negative diagnoses [9,26]. In the present study, if clinical assessment was the only means of evaluation, all repairs would have been considered healed. Nevertheless, MRI at 12 months showed the presence of a meniscal cyst in one (asymptomatic and satisfied) patient, forcing us to consider healing rate. Magnetic resonance imaging (MRI) has been proven as a fairly reliable, non-invasive, imaging modality towards accurate diagnosis, although it is certainly not superior to second-look arthroscopy [9,26] or MR arthrogram [26]. In a study of 89 patients who underwent second-look arthroscopy following meniscal repair and ACL reconstruction, the healing rates for the medial and the lateral meniscus were 90.8% and 75.0%, respectively (with no significant difference), corresponding to 77 patients. In the same study, if clinical examination alone were to be used, only 63 patients would have been considered to have a healed meniscal repair. MRI sensitivity, accuracy and specificity ranged widely between five sequences, suggesting that a combination of sequences could improve the diagnostic value of this imaging modality [26]. However, patients with anterior horn injuries, either medial or lateral, were not included in the aforementioned study.

The most reliable technique to assess meniscus healing is arthroscopy [26]. MRI is not considered the gold standard because signal changes persist for a long time and often do not correlate with clinical symptoms [27]. Changes in MRI may occur even in asymptomatic knees. A high rate of false-positive MRI findings when diagnosing a lack of meniscal healing has been reported, reaching 38% in asymptomatic patients [28], with commonly used criteria [21]. The scar at the repaired site quite often expresses a grade 3 signal that can be seen as evidence of an unhealed meniscus. If clinical signs are present, then positive predictive value increases [26]. Thus, when the healing status is uncertain, magnetic resonance arthrography (MRA) might be a better choice than MRI. In our series, one patient had grade 3 signal and one had a postoperative meniscal cyst at the site of repair, with excellent patient-reported scores and no clinical signs or symptoms. In the study by Pujol et al with long-term follow-up with MRI, the mean subjective IKDC and Lysholm scores of the patients with no abnormal signal on MRI were 94.7 and 99, the mean subjective IKDC and Lysholm scores of the patients with vertical abnormal signals on MRI were 99 and 100 and the mean subjective IKDC and Lysholm scores of the patients with horizontal grade 3 abnormal signals on MRI were 83.9 and 99, respectively. These abnormal vertical and/or horizontal hypersignals present on MRI 10 years after arthroscopic all-inside meniscal repair, had no subjective or objective clinical significance [28]. The high healing rates in the present study are owed to isolated meniscal biologic potential and biomechanical advantage of lesser loading.

Tears of the anterior horn of the lateral meniscus have been shown to significantly increase tibiofemoral contact pressures in both the medial and lateral compartments of the knee [29]. Furthermore, repair of these tears has been reported to restore contact pressures to normal values. Therefore, surgical repair of meniscal lesions is indicated whenever possible [24]. On the other hand, optimal treatment of the macerated, degenerative-like tears in the anterior horn remains unclear, due to poorer patient-reported outcomes and questionable mechanical integrity of meniscal repair [18]. Latest research does not support the previous hypothesis that younger age at the time of meniscal repair would result in fewer failures [11]. Recent studies do not support correlation between age and an increased risk for reoperation [19]. One possible explanation is lower physical activity level of patients over 40 or more conservative case selection by the surgeon. A previous study has indicated that even a partially healed meniscal repair might protect against future osteoarthritis [30], suggesting that leaving an asymptomatic, potentially partially healed repaired meniscus with an inconclusive MRI, appears preferable to resection.

Limitations

Limitations of this study are its retrospective nature, the small number of patients without recorded biometric information, the very specific population included (with isolated anterior horn tears), the use of MR imaging instead of the more reliable (but invasive) second-look arthroscopy in order to assess healing and the lack of a control group for comparison.

## Conclusions

Outside-in is a reliable technique to repair meniscal anterior horn tears, both medially and laterally. We report high healing rates and high scores of patient satisfaction in young, active patients, owing to isolated meniscal biologic potential and good surgical technique.
